# Explaining the Efficacy of an Internet-Based Behavioral Activation Intervention for Major Depression: A Mechanistic Study of a Randomized-Controlled Trial

**DOI:** 10.32872/cpe.5467

**Published:** 2021-09-30

**Authors:** Zhongfang Fu, Huibert Burger, Retha Arjadi, Maaike H. Nauta, Claudi L. H. Bockting

**Affiliations:** 1 Department of Psychiatry, Amsterdam University Medical Centers, location AMC, University of Amsterdam, Amsterdam, The Netherlands; 2 Department of General Practice and Elderly Care Medicine, University Medical Center Groningen, University of Groningen, Groningen, The Netherlands; 3 Faculty of Psychology, Atma Jaya Catholic University of Indonesia, Jakarta, Indonesia; 4 Department of Clinical Psychology and Experimental Psychopathology, University of Groningen, Groningen, The Netherlands; 5 Centre for Urban Mental Health, University of Amsterdam, Amsterdam, The Netherlands; Philipps-University of Marburg, Marburg, Germany

**Keywords:** psychological interventions, working mechanisms, behavioral activation, depression, internet-based intervention, lay counselors

## Abstract

**Background:**

Behavioral activation is an effective treatment for depression that is theorized to facilitate structured increases in enjoyable activities that increase opportunities for contact with positive reinforcement; to date, however, only few mechanistic studies focused on a standalone intervention.

**Method:**

Interventions using internet-based behavioral activation or psychoeducation were compared based on data from a randomized-controlled trial of 313 patients with major depressive disorder. Activation level and depression were measured fortnightly (baseline, Weeks 2, 4, 6, 8, 10), using the Patient Health Questionnaire-9 and the Behavioral Activation for Depression Scale-Short Form, respectively. Analysis was performed to determine if a change in activation level mediated treatment efficacy.

**Results:**

Latent growth modeling showed that internet-based behavioral activation treatment significantly reduced depressive symptoms from baseline to the end of treatment (standardized coefficient = −.13, p = .017) by increasing the rate of growth in the activation level (mediated effect estimate = −.17, 95% CI [−.27, −.07]. Results from mixed effects and simplex models showed that it took 4 weeks before mediation occurred (i.e., a significant change in activation that led to a reduction in depressive symptoms).

**Conclusion:**

Activation level likely mediated the therapeutic effect of behavioral activation on depression in our intervention. This finding may be of significant value to clinicians and depressed individuals who should anticipate a 4-week window before seeing a prominent change in activation level and a 6-week window before depressive symptomatology reduces. Future research must consolidate our findings on how behavioral activation works and when mediation occurs.

## Highlights

- Activation level mediates depression outcomes in an 8-week internet-based behavioral intervention.
- Internet-based behavioral activation appeared to work by changing the level of activation at Week 4 and reducing depressive symptoms over the next 2 weeks.
- Internet-based treatment requires patience and perseverance from clinicians and patients.

## Background

Depression is a prevalent and disabling mental health condition characterized by sadness and lack of interest (American Psychiatry Association, 2015). Behavioral activation is well-established as an effective treatment (Cuijpers, Van Straten, & Warmerdam, 2007; Stein, Carl, Cuijpers, Karyotaki, & Smits, 2021) and as a standalone therapy in relevant clinical guidelines (National Collaborating Centre for Mental Health [UK], 2010). It is also considered a cost-effective therapy that can be delivered easily and disseminated in a range of formats (Arjadi et al., 2018; Carlbring et al., 2013). However, more research is needed to clarify uncertainties about how behavioral activation exerts its clinical effects (Janssen et al., 2020).

Rooted in behavioral frameworks, the theory underpinning behavioral activation conceptualizes depression as the result of low levels of (response-contingent) positive reinforcement: the consequences of environmental interaction that increase the likelihood of a given behavior (Ferster, 1973, 1981; Lazarus, 1972; Lewinsohn, 1974). The theory posits that a lack of this positive reinforcement can result in decreased behavioral activation or withdrawal from the environment, which precipitates depression (Manos, Kanter, & Busch, 2010). Therefore, actively engaging in behavioral activation can help to break the negative cycle of depression by promoting meaningful and adaptive engagement in life (Martell, Dimidjian, & Herman-Dunn, 2013). This strong theoretical basis allows for changes in levels of activation and avoidance (i.e., the activation level) to be evaluated as the hypothesized mediator of change in depressive symptoms during treatment (Curry & Meyer, 2016). However, two research gaps remain. First, contrasting starkly with research into cognitive processes, there is limited empirical evidence of activation level as a potential mediator (Lemmens, Müller, Arntz, & Huibers, 2016; Moreno-Peral et al., 2020). Second, mediators have rarely been examined in randomized-controlled trials (RCTs) of behavioral activation as a standalone treatment (Janssen et al., 2020). Further study is needed to correct this lack of mechanistic research into mediation processes.

Most research into behavioral activation has investigated it as a component of cognitive behavior therapy (e.g., van Luenen, Kraaij, Spinhoven, Wilderjans, & Garnefski, 2019), for which the underlying theoretical assumption differs, suggesting instead that behavioral change helps to improve symptoms through cognitive restructuring. To date, ten studies have examined activation level for the treatment of depression (Dimidjian et al., 2017; Forand et al., 2018; Gaynor & Harris, 2008; Nasrin, Rimes, Reinecke, Rinck, & Barnhofer, 2017; Richards et al., 2017; Rovner et al., 2014; Santos et al., 2019; Silverstein et al., 2018; van Luenen et al., 2019; Weidberg, González-Roz, García-Fernández, & Secades-Villa, 2021). Among these, four investigated a standalone behavioral activation intervention, producing inconsistent results, and none assessed both depression and activation during treatment, precluding mediation analyses. The inconsistent findings likely result from clinical heterogeneity and a failure to meet specific methodological requirements, such as using an RCT design, examining variables of interest longitudinally to assess temporal ordering, and being sufficiently large to ensure robust statistical analyses (Curran et al., 2010; Kazdin, 2007; Lemmens et al., 2016). Studies assessing the activation level as a mediator of depression treatment have not complied with all these requirements (Janssen et al., 2020), with some adopting small samples (e.g., <40 per trial arm) (Gaynor & Harris, 2008) and others using too few repeat observations (e.g., <3) (Richards et al., 2017; Weidberg et al., 2021) or no control group (e.g., Santos et al., 2019). Thus, adequately powered trials of standalone behavioral activation interventions for depression are needed to clarify the extent to which the activation level mediates treatment outcomes.

Our group has previously conducted an RCT for an internet-based intervention involving a large sample of patients with major depressive disorder treated by behavioral activation under the guidance of lay counselors (intervention) compared with psychoeducation (controls) (Arjadi et al., 2018). In that study, we concluded that, after 10 weeks, patients in the intervention group reported significantly fewer depressive symptoms (effect size, 0.24) and had a 50% higher chance of remission than those in the control group. Crucially, this study complied fully with the methodological requirements of mechanistic research into mediation processes. In the present study, we therefore aimed to use data from that study to demonstrate that the activation level mediates the relationship between treatment with behavioral activation and improved depression. This was considered achievable if we could demonstrate two criteria (Kazdin, 2007; MacKinnon, 2008). First, that the treatment condition correlated with changes in the activation level, which in turn, correlated with changes in depressive symptoms and was conditional on treatment allocation (Criterion 1). Second, that the change in activation level produced the change in depressive symptoms, and not vice versa (i.e., temporal ordering; Criterion 2).

## Materials and Method

### Design

This study reports on a post-hoc analysis of an earlier two-group RCT of an internet-based behavioral activation program for patients with major depressive disorders (*N* = 313). Details of the original RCT are reported elsewhere (Arjadi et al., 2018). All assessments were completed on the Qualtrics survey platform and administered at baseline and every 2 weeks thereafter up to the main post-treatment evaluation at Week 10 (endpoint), with follow-up at 12 and 24 weeks after baseline. For the purposes of the current study, depression and activation level were examined fortnightly at baseline and at Weeks 2, 4, 6, 8, and 10.

### Participants and Randomization

In total, 313 participants were included and randomized into the treatment (*n* = 159) and control (*n* = 154) groups (see Arjadi et al., 2018, for a detailed flowchart). The baseline characteristics we comparable in each group, as presented in Table 1, indicating successful randomization. Participants were recruited via online self-referral. Eligible participants were aged ≥16 years, scored ≥10 on the Patient Health Questionnaire-9 (PHQ-9), and had a principal diagnosis of major depressive disorder or persistent depressive disorder defined according to the Diagnostic and Statistical Manual of Mental Disorders, Fifth Edition. Diagnosis was by semi-structured diagnostic interview (SCID-5) (First et al., 2015). Participants with current substance use disorder, current or previous manic or hypomanic episodes, psychotic disorder, or acute suicidality were excluded, as were those receiving psychological interventions.

**Table 1:** Descriptive Statistics of Baseline Demographic Characteristics

Demographic information	GAF (*n* = 159)^a^	PE (*n* = 154)^a^
Age (*M*, *SD*)	24.5 (4.9)	24.5 (5.2)
Sex		
Female	128	125
Male	31	29
Current PTSD		
Yes	22	30
No	137	124
Education		
Above bachelor	89	81
Others	70	73
Living area		
Urban	93	96
Others	67	58
Socioeconomic class		
Low	32	27
Middle	98	100
High	29	27
Ethnicity		
Java	69	64
Tionghoa	30	18
Sunda	21	22
Others	39	40

^a^Note that all patients were in a depressive episode. Abbreviations: GAF = Guided Act-and-Feel-Indonesia; PE = Psychoeducation; PTSD = post-traumatic stress disorder; *SD* = standard deviation.

Eligible participants were allocated (1:1) by a research assistant in a random permuted block design stratified by sex and depression severity (score 10–14 or ≥15 on the PHQ-9) via a web-based program. Current depressive episodes and post-traumatic stress disorder were assessed by clinical diagnostic interview conducted by trained clinical interviewers who were required to hold at least a bachelor’s degree in psychology.

### Treatments

#### Intervention Group: Guided Act-and-Feel-Indonesia (GAF-ID)

Participants in the intervention group received an internet-based behavioral activation intervention (the GAF-ID) supported by lay counselors. The intervention program was adapted from an online intervention for behavioral activation based on Lewinsohn’s (1974) theory of depression. The original program was published in Dutch (Doe en Voel; Bockting & Van Valen, 2015) and was translated to Bahasa Indonesian. The GAF-ID program was delivered using an online platform in eight structured modules delivered weekly. Each module was expected to be completed online in 30–45 minutes. The intervention group was guided and supported by lay counselors who were supervised by a licensed clinical psychologist. A detailed description of the guidance and support is available elsewhere (Arjadi et al., 2018).

#### Control Group: Online Psychoeducation

Participants in the control group were given access to another online platform from which they could find basic psychoeducation on depression and brief tips on coping with depression in general. This information was distilled from the psychoeducation module of the GAF-ID program, but no guidance or support was provided.

### Measures

Demographic information was collected at baseline, including age, gender, ethnicity, education (above bachelor/other), living area (urban/other), and socioeconomic class. The latter was determined by monthly expenditure in Indonesian rupiah (IDR): low, <1 million; middle, 1–5 million; and high, >5 million. In addition, the PHQ-9 and Behavioral Activation for Depression Scale-Short Form (BADS-SF) were completed fortnightly.

#### Patient Health Questionnaire-9 Item Version

The PHQ-9 is a 9-item self-report questionnaire in which participants rate how they felt during the previous two weeks (e.g., “Feeling tired or having little energy”). Each question is scored 0 to 3 (0 = *not at all*, 1 = *several days*, 2 = *more than half the days*, and 3 = *nearly every day*). Sum scores range from 0 to 27, with higher scores representing higher levels of depression. The PHQ-9 has acceptable validity and reliability (Carroll et al., 2020), and the Cronbach’s alphas in the current study ranged from .78 to .87 at the different assessments.

#### Behavioral Activation for Depression Scale-Short Form

The BADS-SF is a 9-item self-report questionnaire that measures changes in activation and avoidance in the previous week (e.g., “There were certain things I needed to do that I didn’t do”). Each question is scored 0 to 6 (0 = *not at all*, 6 = *completely*). Items 1, 6, 7, and 8 are reverse-coded. Sum scores can range from 0 to 54, with higher scores representing higher activation. The validity and reliability of BADS-SF have been established (Manos, Kanter, & Luo, 2011), and the Cronbach’s alphas in the current study ranged from .78 to .88 at different assessments.

### Data Analysis

#### Mixed Effects Model to Compare Mean Depression and Activation Levels

Mixed effects models were used to inspect how treatment influenced activation level and depression at each time point. Baseline and follow-up measures were treated as response variables. Missing values were imputed by multiple imputation, including treatment allocation and all PHQ-9 and BADS-SF assessments in the predictor matrix. Given that the functional form of the mean responses during treatment can be difficult to anticipate, time was specified as a class effect in an unstructured manner. The contrasts between treatment groups at each time point were obtained by comparing the least squares means of the variables of interest. Mixed effect analyses were conducted using the *nlme* R package (Pinheiro, Bates, DebRoy, Sarkar, & R Core Team, 2020), and for multiple imputations, we used the *mice* R package (van Buuren & Groothuis-Oudshoorn, 2011).

#### Mediation Analyses Using Latent Growth and Simplex Mediation Models

Mediation analyses were based on latent growth models to address criterion 1 (MacKinnon, Cheong, & Pirlott, 2012) and simplex mediation models to address criterion 2 (Goldsmith et al., 2018) in a structural equation model framework.

We refer to the path estimating the relationship between treatment allocation (T) and activation level (M) as the *a* path and refer to the path between activation level and depression (Y) as the *b* path. The direct effect from treatment allocation to depression is noted as the *c* path, after accounting for M as *c′*. The product of *a × b* coefficients method was used to indicate the indirect effect (Goldsmith et al., 2018). Coefficients were provided based on a completely standardized solution, and the confidence intervals of *a* × *b* were estimated by bootstrapping (1,000 times). A mediated effect was deemed statistically significant if the 95% confidence interval (95% CI) did not cross zero.

Latent growth model analyses were performed in three steps to model the relationship between treatment and the growth trajectories of activation and depression (Cheong, MacKinnon, & Khoo, 2003). First, to investigate the shape of the growth trajectories for depression and activation, unconditional growth models were built. Second, to examine if the growth rates of depression and activation differed by treatment condition, two conditional models were constructed with the treatment conditions. Third, to assess the indirect effect of treatment allocation on the outcome, via the mediator (activation level), we combined the two conditional growth models into a parallel process growth model. In this, the path coefficients (*a*, *b*, *c*, and *c′*) of the mediation model were estimated and the contributions of baseline characteristics as covariates were examined (e.g., sex, ethnicity, urban/rural, socioeconomic status, post-traumatic stress disorder, and education level).

A simplex mediation model was then adopted to determine if there was temporal ordering. This was achieved by evaluating whether a prior activation level was associated with the level of depression at a subsequent measurement. We specified models as either a lagged *b* path (activation affects depression at adjacent time points) or a contemporaneous *b* path (activation affects depression at the same time point). We added treatment allocation as a time-invariant antecedent variable to predict depression and activation level at each time point. Autoregressive and cross-lagged effects were constrained to be equal over time (Goldsmith et al., 2018). To assess the timing of the potential mediation process, *a* paths were freely estimated. In addition, to evaluate the extent to which prior depression influenced the subsequent activation level, we reversed the position of depression and activation level in a supplementary analysis (see Supplementary Materials).

**Figure 1:**
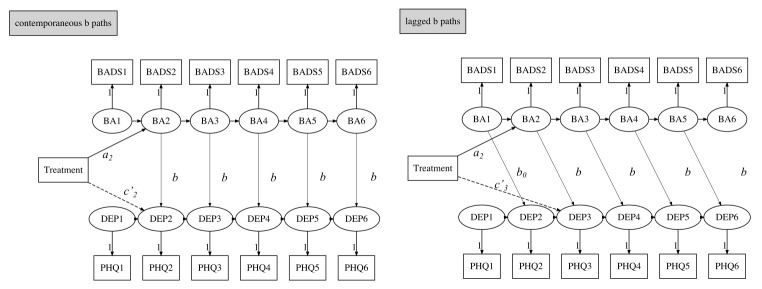
Example Diagram of Simplex Models for Mediation With Contemporaneous *b* Paths (Right Side) and Lagged *b* Paths (Left Side) With Depression at Third Timepoint (Week 6) as Outcome *Note.* Abbreviations: *a*_2_ = parameter estimated coefficient from treatment to Week 4 behavioral activation; *b* = parameter estimated coefficient from mediator to outcome; *b*_0_ = parameter estimated coefficient from baseline mediator to Week 2 depression; BA, behavioral activation; BADS(1, 2, 3, 4, 5, 6) = Behavioral activation of depression scale-Short form (baseline and 2, 4, 6, 8, 10 weeks, respectively); *c′*_2_, *c′*_3_ = parameter estimated coefficient from treatment to Week 4, 6 depression after controlled for intermediate behavioral activation; PHQ(1, 2, 3, 4, 5, 6) = Patient Health Questionnaire-9 items (baseline and 2, 4, 6, 8, 10 weeks, respectively).

The time-specific indirect effect was estimated using a series of product terms to indicate the possible timing of the putative mediator taking effect. Figure 1 shows an example simplex model with lagged *b* paths: for the third time point, depression Y_3_ (i.e., Week 4 depression), one indirect effect of treatment could be T→ M_2_ →Y_3_. Calculation was performed as *a*_2_ × *b*_23_, where the subscripts indicated direction (e.g., the coefficient *a*_2_ was the effect to activation at Point 2, and *b*_23_ was the effect from activation at Point 2 to depression at Point 3, and all *b* paths were considered equal). A significant result could suggest a lagged mediation effect from Week 2 activation (M_2_) to Week 4 depression (Y_3_). The overall indirect effect in the model for Y_3_ was the sum of all time-specific indirect effects estimated by the products of the parameters that estimated the paths between T and Y_3_ and passed through the mediator. Coefficient *a* at baseline (i.e., *a*_1_) was fixed at zero because treatment had not been implemented at this time.

Data were assumed to be missing at random or completely at random (Graham, 2009), so we used a full-information maximum likelihood estimation in the structural equation modeling analysis. Participants who had at least one measurement for depression were retained in the model and analysis performed on an intention-to-treat basis. Model fit was assessed by the comparative fit index (CFI), Tucker–Lewis index (TLI), root mean squared error of approximation (RMSEA), and standardized root mean square residual (SRMR). We used established guidelines of acceptable fit, requiring that the CFI and TLI should exceed 0.90–0.95, that the RMSEA should not exceed 0.06–0.10, and that the SRMR should not exceed 0.08. All structural equation modeling analyses were performed in Mplus 8.3 (Muthén & Muthén, 2019).

## Results

A full overview of the levels of activation and depression at each measurement is presented in Table 2.

**Table 2:** Means and Standard Deviations of PHQ-9 and BAD-SF for Each Group at Each Assessment

Measure	GAF-ID			PE		
	Missing	Means	*SD*	Missing	Means	*SD*
Depression (PHQ-9)						
Week 0 (Baseline)	0	17.92	5.39	0	18.01	5.05
Week 2	21	12.04	6.05	2	12.81	5.97
Week 4	33	10.53	6.04	10	11.33	6.01
Week 6	31	9.79	5.80	8	11.18	5.85
Week 8	43	9.07	6.22	11	10.48	6.12
Week 10 (Endpoint)	39	8.50	5.75	9	10.83	6.21
Behavioral activation (BADS-SF)						
Week 0 (Baseline)	0	16.67	6.72	0	16.38	6.29
Week 2	21	19.59	6.75	2	18.68	6.64
Week 4	33	23.22	7.32	10	19.93	6.87
Week 6	31	24.11	7.94	8	20.57	7.61
Week 8	43	24.93	8.06	11	22.22	7.72
Week 10 (Endpoint)	39	24.12	7.37	9	20.73	7.45

*Note.* Abbreviations: BADS-SF = Behavioral Activation for Depression Scale – Short Form; GAF = Guided Act-and-Feel-Indonesia; PE = Psychoeducation; PHQ-9 = Patient Health Questionnaire-9; *SD* = standard deviation.

Each fortnightly assessment was completed by at least 83% of the sample, but 17.5% of all data points were missing in the GAF-ID group versus 4.3% in the control group. Participants in both groups had at least 4 data points (83.6% for the GAF-ID group and 95.4% for the control group). The main reasons for dropout at Week 10 were “no time” (18 in the GAF-ID group) and “no improvement” (12 in GAF-ID group and 6 in the control group).

### Mixed Effects Model: Differences of Depression and Activation Level

Treatment allocation had significant effects on depression (*p* < .001) and activation (*p* < .001) across all included time points. As shown in Table 3, the mean differences in activation and depression increased over time between the treatment and control groups, reaching statistical significance from Week 4 (Assessment 3) for activation and Week 6 (Assessment 4) for depression.

**Table 3:** Means Difference of Depression and Activation Between Treatment and Control Groups Over Time (Unstructured Time Model)

Time point	LSMD	*SE*	95% CI	*p value*
Behavioral Activation (BADS-SF)				
Week 0 (Baseline)	0.30	0.74	[−0.77, 1.36]	.688
Week 2	0.70	0.77	[−0.46, 1.87]	.360
Week 4	3.47	0.94	[1.72, 5.21]	**< .001**
Week 6	3.41	1.01	[1.39, 5.42]	**.002**
Week 8	2.86	0.96	[1.05, 4.63]	**.004**
Week 10 (Endpoint)	3.36	0.89	[1.82, 4.91]	**< .001**
Depression (PHQ-9)				
Week 0 (Baseline)	−0.08	0.59	[−0.77, 0.60]	.890
Week 2	−0.61	0.69	[−1.55, 0.33]	.379
Week 4	−0.97	0.72	[−1.97, 0.04]	.178
Week 6	−1.41	0.68	[−2.31, −0.50]	**.039**
Week 8	−1.76	0.74	[−0.68, −2.84]	**.019**
Week 10 (Endpoint)	−2.59	0.71	[−3.56, −1.61]	**< .001**

*Note.* Abbreviations: BADS-SF = Behavioral activation for depression scale-short form; CI = confidence interval; LSMD = least squares mean difference; PE = Psychoeducation; PHQ-9 = Patient Health Questionnaire-9; *SE* = standard error.

### Latent Growth Model for Mediation

#### Unconditional Growth Model

Model fit indices, as shown in Table 4, were acceptable. The RMSEA for the model of depression was higher than that of activation level, suggesting that the variance in depression could be explained by a potential covariate (e.g., treatment).

**Table 4:** Fit Indices of Latent Growth Models

Model	CFI	TLI	RMSEA (90%CI)	SRMR
Depression (unconditional model)	0.96	0.94	0.11 [0.08, 0.14]	0.07
Treatment–Depression	0.96	0.94	0.09 [0.07, 0.12]	0.06
BA (unconditional model)	0.99	0.99	0.04 [0, 0.08]	0.04
Treatment–BA	0.99	0.99	0.04 [0, 0.07]	0.04
Treatment–BA–Depression	0.97	0.96	0.05 [0.04, 0.07]	0.05

*Note.* Abbreviations: BA = Behavioral activation; CFI = comparative fit index; CI = confidence interval; RMSEA = root mean squared error of approximation; SRMR = standardized root mean square residual; TLI = Tucker–Lewis index.

#### Conditional Growth Models: The Effect of Treatment

The fitness of both conditional models appeared acceptable (Table 4). The GAF-ID group showed a larger increase in activation (standardized coefficient = .27, *p* < .001) and a larger reduction in depression compared with the control group (standardized coefficient = −.13, *p* = .017). This confirmed that treatment was efficacious in producing a difference in trajectories between the treatment and control groups.

#### Parallel Process Growth Models: The Mediation Effect

**Figure 2:**
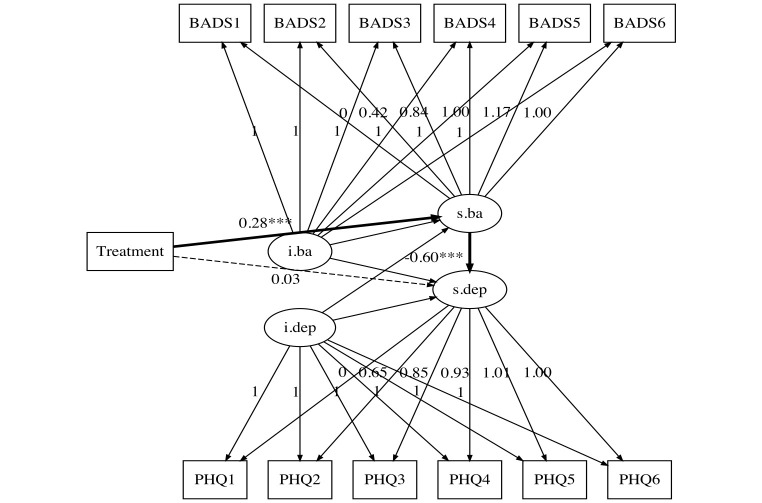
Parallel Process Latent Growth Model of Depression and Activation Level Conditioned on Treatment Groups *Note.* Rectangles denote observed variables, and ellipses denote latent variables. Bolded arrows indicated the significant prediction from treatment to growth of activation, growth of activation to growth of depression. Dashed arrow indicated the insignificant prediction from treatment to growth of depression. Abbreviations: BADS(1, 2, 3, 4, 5, 6) = Behavioral activation of depression scale-Short form (baseline and 2, 4, 6, 8, 10 weeks, respectively); i.dep = intercept growth factor of depression; i.ba = intercept growth factor of behavioral activation; PHQ(1, 2, 3, 4, 5, 6) = Patient Health Questionnaire-9 items (baseline and 2, 4, 6, 8, 10 weeks, respectively); s.ba = slope growth factor of behavioral activation; s.dep = slope growth factor of depression.

Model fit of the parallel process growth model was acceptable (Figure 2). Factor loadings of the slope growth factor indicating the predicted trajectory of depression and activation are presented in Table 5.

Consistent with the plotted growth trajectory for depression based on data for the whole sample (see Figure 3a), there was a sharp decrease (0.65 unit) in depressive symptoms from the second week. The reduction in depression continued, reaching a trough at Week 8 that persisted to Week 10 (endpoint). A slightly different pattern was observed for the trajectory of the activation level. As shown in Figure 3b and Table 5, activation increased by 0.42 units after the second week of treatment, peaking at Week 8 before decreasing slightly at Week 10 (endpoint).

**Figure 3a:**
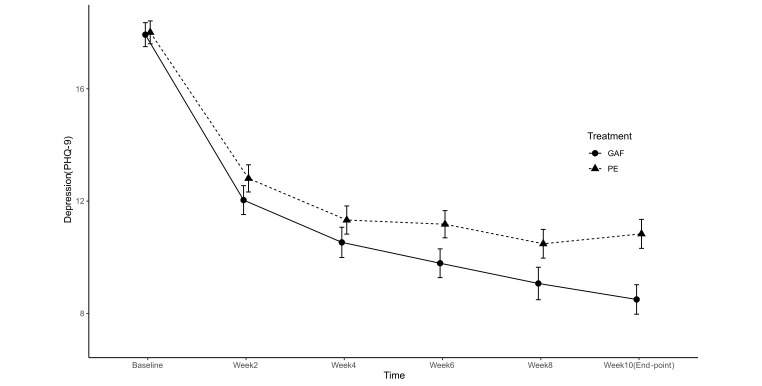
Trajectories of Depression (PHQ-9) Across Measurements in Treatment (GAF) and Control (PE) Groups *Note.* GAF = Guided Act and Feel treatment; PE = Psychoeducation.

**Figure 3b:**
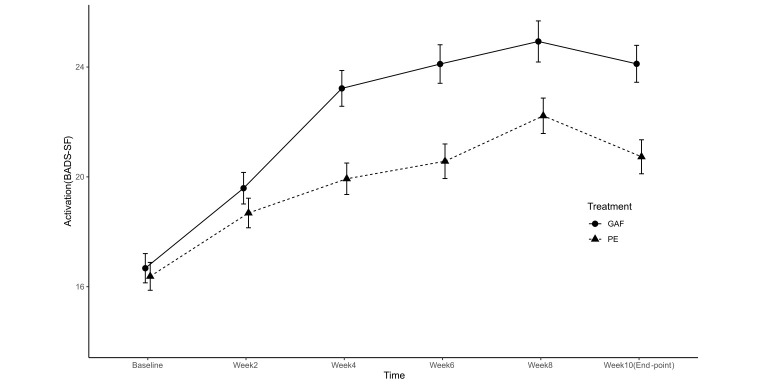
Trajectories of Activation (BADS-SF) Across Measurements in Treatment (GAF) and Control (PE) Groups *Note.* GAF = Guided Act and Feel treatment; PE = Psychoeducation.

**Table 5:** Growth Factor Loadings for Intercept and Slope Factors in the Parallel Latent Growth Models for Depression and Activation Level

Time point	Depression (PHQ-9)		Behavioral Activation (BADS-SF)	
	Intercept	Slope	Intercept	Slope
Week 0 (Baseline)	1	0	1	0
Week 2	1	0.65	1	0.42
Week 4	1	0.85	1	0.84
Week 6	1	0.93	1	1.00
Week 8	1	1.01	1	1.17
Week 10 (Endpoint)	1	1.00	1	1.00

*Note.* Abbreviations: BADS-SF = Behavioral activation for depression scale-short form; PHQ-9 = Patient Health Questionnaire-9.

Treatment condition (GAF-ID or control) was significantly associated with the slope factor of activation level (path *a*, standardized coefficient = 0.28, *p* < .001), which in turn was associated with the slope factor of depression (path *b*, standardized coefficient = −0.60, *p* < .001). After accounting for the growth trajectory of the activation level, the prediction that treatment affected depression was no longer significant (path *c*′, standardized coefficient = 0.03, *p* = .483). Table 6 shows that the estimated mediated effect (*a × b* product) was standardized as −0.17, 95% CI [−0.27, −0.07], *p* = .001. After adding the baseline characteristics as covariates, model fit was similar, CFI = 0.97, TLI = 0.96, RMSEA = 0.04, 90% CI [0.03, 0.05], and SRMR = 0.05. The estimated mediated effect in this model was similar to that in the model without baseline characteristics as covariates, standardized estimate = −0.15, 95% CI [−0.25, −0.08], *p* < .001.

**Table 6:** Regression Coefficients of Mediational Parallel Process Growth Models

Model	Standard coefficient	*SE*	*p* value
Conditional Models			
Treatment–Depression	−0.13	0.06	**.017**
Treatment– BA	0.27	0.06	**< .001**
Parallel process model			
Treatment–BA (*a* path)	0.28	0.06	**< .001**
BA–Depression (*b* path)	−0.60	0.08	**< .001**
Treatment–Depression (*c′* path)	0.03	0.05	.483
*a* × *b* product	−0.17	0.05	**.001**

*Note.* Abbreviations: BA = Behavioral activation; *SE* = standard error.

### Time-Specific Mediation Effect in the Simplex Models

For the simplex models with activation level as a mediator, fit indices with a contemporaneous *b* path were adequate, CFI = 0.96, TLI = 0.95, RMSEA = 0.06, 90% CI [0.05, 0.08], and SRMR = 0.07. Table 7a shows that the contemporaneous indirect effect reached significance from Week 6. Table 7b summarizes the results with only significant lagged indirect paths, showing that the paths all passed through M_3_ (i.e., activation level at Week 4) to influence either contemporary depression or subsequent mediators (M*_n_*), and ultimately, later depression. Fit indices of the simplex mediation model with the lagged *b* path were adequate, CFI = 0.95, TLI = 0.94, RMSEA = 0.07, 90% CI [0.06, 0.08], and SRMR = 0.08. As shown in Table 7b, the indirect effect reached significance from Week 6 onwards. As with the contemporaneous *b* paths, M_3_ was the only mediator to be passed through during the treatment.

**Table 7a:** Simplex Model With Contemporaneous B Paths for Activation Level as a Mediator

Simplex for mediation with contemporaneous *b* path		*SE*	*p*	95% CI	
Time-specific outcome / Significant Paths and Effect of treatment	Standardized estimate			*LL*	*UL*
Week 2 Depression (Y_2_)					
Total effect	−0.05	0.05	.320	−0.17	0.06
Indirect effect	−0.01	0.01	.379	−0.03	0.01
Week 4 Depression (Y_3_)					
Total effect	−0.08	0.06	.189	−0.20	0.05
Indirect effect	−0.09	0.04	.035	−0.19	−0.001
T→M_3_→Y_3_	−0.04	0.02	.006	−0.08	−0.01
Week 6 Depression (Y_4_)					
Total effect	−0.13	0.06	.028	−0.25	−0.004
Indirect effect	−0.12	0.05	.016	−0.22	−0.01
T→M_3_→Y_3_→Y_4_	−0.03	0.01	.005	−0.06	−0.01
T→M_3_→M_4_→Y_4_	−0.04	0.02	.006	−0.08	−0.02
Week 8 Depression (Y_5_)					
Total effect	−0.15	0.06	.012	−0.27	−0.02
Indirect effect	−0.14	0.05	.003	−0.25	−0.04
T→M_3_→Y_3_→Y_4_→Y_5_	−0.02	0.01	.004	−0.04	−0.01
T→M_3_→M_4_→Y_4_→Y_5_	−0.03	0.01	.004	−0.05	−0.01
T→M_3_→M_4_→M_5_→Y_5_	−0.04	0.01	.005	−0.07	−0.01
Week 10 Depression (Endpoint, Y_6_)					
Total effect	−0.22	0.06	< .001	−0.34	−0.09
Indirect effect	−0.16	0.05	.001	−0.26	−0.06
T→M_3_→Y_3_→Y_4_→Y_5_→Y_6_	−0.02	0.01	.004	−0.03	−0.01
T→M_3_→M_4_→Y_4_→Y_5_→Y_6_	−0.02	0.01	.004	−0.03	−0.01
T→M_3_→M_4_→M_5_→Y_5_→Y_6_	−0.03	0.01	.004	−0.05	−0.01
T→M_3_→M_4_→M_5_→M_6_→Y_6_	−0.04	0.01	.005	−0.06	−0.01

*Note.* Only significant paths are shown to save space. Abbreviations: M_3_, M_4_, M_5_ = mediator measurements (taken at Weeks 4, 6, and 8, respectively); *SE* = standard error; T = Treatment allocation (treatment group = 1, control group = 0); Y_2_, Y_3_, Y_4_, Y_5_, Y_6_ = outcome measurements (taken at Weeks 2, 4, 6, 8, and 10, respectively).

**Table 7b:** Simplex Model With Lagged b Paths for Activation Level as a Mediator

Simplex model for mediation with lagged *b* path		*SE*	*p*	95% CI	
Time-specific outcome / Significant Paths and Effect of treatment	Standardized estimate			*LL*	*UL*
Week 4 Depression (Y_3_)					
Total effect	−0.08	0.06	.187	−0.21	0.05
Indirect effect	−0.05	0.05	.269	−0.15	0.03
Week 6 Depression (Y_4_)					
Total effect	−0.13	0.06	.025	−0.25	−0.01
Indirect effect	−0.11	0.05	.033	−0.22	0.001
T→M_3_→Y_4_	−0.04	0.02	.01	−0.07	−0.01
Week 8 Depression (Y_5_)					
Total effect	−0.16	0.06	.01	−0.28	−0.02
Indirect effect	−0.15	0.05	.003	−0.26	−0.04
T→M_3_→Y_4_→Y_5_	−0.03	0.01	.008	−0.05	−0.01
T→M_3_→M_4_→Y_5_	−0.04	0.02	.01	−0.07	−0.01
Week 10 Depression (Endpoint,Y_6_)					
Total effect	−0.22	0.06	< .001	−0.35	−0.09
Indirect effect	−0.15	0.05	.002	−0.26	−0.04
T→M_3_→Y_4_→Y_5_→Y_6_	−0.02	0.01	.007	−0.04	−0.01
T→M_3_→M_4_→Y_5_→Y_6_	−0.03	0.01	.008	−0.05	−0.01
T→M_3_→M_4_→M_5_→Y_6_	−0.04	0.01	.009	−0.07	−0.01

*Note.* Only significant paths are shown to save space. Abbreviations: M_3_, M_4_, M_5_ = mediator measurements (taken at Weeks 4, 6, and 8, respectively); *SE* = standard error; T = Treatment allocation (treatment group = 1, control group = 0); Y_2_, Y_3_, Y_4_, Y_5_, Y_6_ = outcome measurements (taken at Weeks 2, 4, 6, 8, and 10, respectively).

For the simplex models with depression as a mediator, the fit indices were acceptable for both contemporary *b* paths, CFI = 0.97, TLI = 0.95, RMSEA = 0.06, 90% CI [0.05–0.08], SRMR = 0.06, and lagged *b* paths, CFI = 0.95, TLI = 0.93, RMSEA = 0.07, 90% CI [0.06–0.09], SRMR = 0.08. None of the significant indirect effect from treatment allocation to activation level at each time point passed through depression, indicating that our intervention works though the impact of activation on depression rather than the other way around. More detailed results are provided in the Supplementary Materials.

## Discussion

In this study of data from a large RCT, we provide evidence that activation level underpinned the clinical response to a guided internet-based intervention for depression. During the 8-week treatment period, we showed that (1) our treatment improved activation levels from Week 4 and reduced depressive symptoms from Week 6, and (2) the activation level acted as a mediator for the change in depressive symptoms.

These findings support the theory that a change in depression is contingent on a change in activation level (e.g., Lewinsohn, 1974). We first confirmed that statistically significant associations existed between treatment allocation, activation, and depression level that were not affected by controlling for baseline characteristics. We further supported this by demonstrating temporal order, evidencing that the significant increase in activation level at Week 4 preceded the significant decrease in depressive symptoms at Week 6. This was strengthened by the lack of a “reverse” effect of depression on the activation level when conditioned on treatment. Together, these findings strongly suggest that the hypothesized mediation process occurred around Week 4.

Our findings are consistent with those of similar randomized studies (e.g., Dimidjian et al., 2017; Nasrin et al., 2017; Santos et al., 2017), but conflict with those presented elsewhere. For example, Richards et al. (2017) observed no mediation effect of activation level in a large RCT comparing behavioral activation and cognitive behavioral therapy, nor did Rovner et al. (2014), when they compared behavioral activation and supportive therapy to prevent depression in older adults. There are a couple of plausible explanations for these incongruencies. First, different control conditions were used, with inactive control groups in the first two (waitlist control or usual obstetric care; similar to ours) (Dimidjian et al., 2017; Nasrin et al., 2017) and active control groups in the latter two (Richards et al., 2017; Rovner et al., 2014). Second, measurements were taken at different times, with previous studies assessing mediation either immediately (Dimidjian et al., 2017; Nasrin et al., 2017) or 4 to 6 months (Richards et al., 2017; Rovner et al., 2014) after completing the intervention. Delaying measurements in this way is less likely to capture significant changes caused by the mediator during treatment.

Two studies have used interventions for depression in which the activation level was examined as a putative mediator, and among these, our findings agree with one and disagree with another. In the research by van Luenen et al. (2019) who adopted a similar intervention timeframe (eight sessions completed in 8–10 weeks), it was concluded that the investigated mediation occurred between Weeks 3 and 5. However, this was not apparent in the research by Forand et al. (2018) in another 10-week internet-based trial of cognitive behavioral therapy for depression, who found that the change in activation from baseline to Week 3 did not predict the subsequent change in depression. This inconsistency could be attributed to the fact that Forand et al. (2018) included another potential mediator (cognitive skills) in their mediation model. If activation level were a proximal process that led to another mediation process, controlling for this specific factor may fail to reveal the activation level as a mediator. It could also be that mediation occurred after Week 3 of the intervention; therefore, a test based on earlier change will not have captured the required period. Nevertheless, although the weight of evidence may be shifting, these inconsistencies point to a requirement for more evidence to confirm the mediational role of activation level.

Regarding missing data, more was missing in the intervention group (17.5%) than in the control group (4.3%). This was presumably because the GAF-ID intervention demanded greater effort to accomplish and because some participants could not afford the time. Alternatively, sending the fortnightly measurements via email separately to monitoring within the intervention may have led to some participants erroneously believing that they had already completed the questionnaires.

Our results help to clarify how internet-based and lay-counselor-guided behavioral activation treatments work. Clinicians can use this new knowledge to prepare patients with depression for a 4- to 6-week lag before a major change occurs in their activation level, and subsequently, their symptoms of depression improve. This may encourage depressed individuals to persevere with treatment when they encounter difficulties increasing activity levels in the first phase of treatment. Clinicians and patients alike can be reassured that persistence with therapy will reduce depressive symptoms and lead to recovery.

The present study has several strengths. First, we used data from a well-powered RCT to ensure that the effect estimates from treatment allocation to activation level and depression could be readily and precisely interpreted as causal. The sample size calculated for the RCT was ample for the current mediation analysis, for which a sample size of at least 100 with at least three repeated observations per individual was considered appropriate (Curran et al., 2010). Second, the fortnightly measures added precision and the low dropout rate (0.20%) contributed to both precision and low risk of bias. Third, we adopted latent growth and simplex mediation modeling to estimate, as precisely as possible, the association between the mediator and depression while controlling for the within-participant change. According to criteria set by Lemmens et al. (2016), our work constitutes a high-quality mediation study.

Some limitations also warrant discussion. Notably, the mediator–outcome relationship could still have been confounded by a third unmeasured variable (e.g., cognition). In addition, we only included a single mediator in our model, limiting us to identifying activation as the mediator. Other working mechanisms correlated with activation level may have mediated part its effect, such as a change in cognition that may have preceded the reduction in depressive symptomatology. Aside from using the SCID-5 to assess unipolar depressive disorder before and after treatment, measurements in the RCT relied on self-reporting every 2 weeks. Thus, the assessments of activation level may not have been objective and may have missed a more nuanced dynamic (Folke et al., 2015). Moreover, lay counselors had no role in assessment of the participants and the effect of change in activation level on depression outcomes was also not assessed by lay counselors and fully independently conducted from these counselors. Therefore, although some bias can never be fully excluded, it is unlikely bias explained the outcomes.

Future research must seek to replicate our findings with different control groups. It should have a more temporally sensitive design (e.g., experience sampling method), more objective measures of activation, and include other variables (e.g., cognitive variables). Such research may also benefit from experimental manipulation of mediator levels (e.g., component analysis) (Emmelkamp et al., 2014) and micro-trials using experimental designs, such as RCTs with temporally sensitive designs (Brouwer et al., 2020; Slofstra et al., 2018), to reach firm (causal) conclusions (Lorenzo-Luaces, Lemmens, Keefe, Cuijpers, & Bockting, 2021).

### Conclusion

This study provides evidence that a change in activation level underpinned the effects of a guided internet-based intervention using behavioral activation to treat depression. In a large-scale RCT, it took 4 and 6 weeks to change activation levels and depressive symptoms, respectively. More studies are still required to support these findings and optimize treatment strategies.

## Supplementary Materials

Detailed results for the mediation examination in simplex models with depression as mediator were provided in the Supplementary Materials (for access see Index of Supplementary Materials below).

10.23668/psycharchives.5092 Supplement 1 Supplementary materials to "Explaining the efficacy of an internet-based behavioral activation intervention for major depression: A mechanistic study of a randomized-controlled trial" [Additional results]

FuZ.
BurgerH.
ArjadiR.
NautaM. H.
BocktingC. L. H.
 (2021). Supplementary materials to "Explaining the efficacy of an internet-based behavioral activation intervention for major depression: A mechanistic study of a randomized-controlled trial"
[Additional results]. PsychOpen. 10.23668/psycharchives.5092
PMC966723536398097
