# Supplementary material for: Explaining the Efficacy of an Internet-Based Behavioral Activation Intervention for Major Depression: A Mechanistic Study of a Randomized-Controlled Trial
Source: Clin Psychol Eur. 2021 Sep 30;3(3):e5467. doi: 10.32872/cpe.5467 (PMC9667235; doi:10.32872/cpe.5467)
Supplement: Supplement 1 [file cpe-03-5467-s01.pdf]

Explaining the Efficacy of an Internet-Based Behavioral Activation Intervention for Major Depression: A Mechanistic Study of a Randomized-Controlled Trial

Zhongfang Fu[1], Huibert Burger[2], Retha Arjadi[3,4], Maaike H. Nauta[4], Claudi L. H. Bockting (https://orcid.org/0000-0002-9220-9244)[*1,5]

[1]Department of Psychiatry, Amsterdam University Medical Centers, location AMC, University of Amsterdam, Amsterdam, The Netherlands

[2]Department of General Practice and Elderly Care Medicine, University Medical Center Groningen, University of Groningen, Groningen, The Netherlands

[3]Faculty of Psychology, Atma Jaya Catholic University of Indonesia, Jakarta, Indonesia

[4]Department of Clinical Psychology and Experimental Psychopathology, University of Groningen, Groningen, The Netherlands

[5]Centre for Urban Mental Health, University of Amsterdam, Amsterdam, The Netherlands

*****Meibergdreef 5, 1105 AZ, The Netherlands. c.l.bockting@amsterdamumc.nl

DOI: https://doi.org/10.32872/cpe.5467

Supplementary materials

Table s1:

Simplex model with lagged b paths for depression as mediator

Simplex for mediation with lagged *b* path					95%CI	
Time-specific outcome	Significant Paths and Effect of treatment	Standardized estimate	SE	*p* value	Lower limit	Upper limit
Week 2 Behavioral Activation (Y_2)	Total effect	0.07	0.07	0.336	-0.06	0.20
	Indirect effect	0	0	1.000	0	0

Week 4 Behavioral Activation (Y_3)	Total effect	0.27	0.07	<.001	0.13	0.40
	Indirect effect	0.06	0.05	0.296	-0.05	0.16
Week 6 Behavioral Activation (Y_4)	Total effect	0.29	0.07	<.001	0.15	0.42
	Indirect effect	0.23	0.06	<.001	0.11	0.35
	T->Y_3->Y_4	0.18	0.06	0.002	0.07	0.29
Week 8 Behavioral Activation (Y_5)	Total effect	0.24	0.07	0.001	0.09	0.38
	Indirect effect	0.26	0.07	<.001	0.13	0.49
	T->Y_3->Y_4->Y_5	0.15	0.05	0.003	0.06	0.26
Week 10 Behavioral Activation (End-point, Y_6)	Total effect	0.30	0.07	<0.001	0.16	0.44
	Indirect effect	0.24	0.07	0.001	0.09	0.38
	T->Y_3->Y_4->Y_5->Y_6	0.14	0.05	0.004	0.05	0.24

Note: Only significant paths were showed in the above table to save space. T = Treatment allocation (treatment group = 1, control group = 0); Y_2, Y_3, Y_4, Y_5, Y_6 = behavioral activation measurement taken at week 2, 4, 6, 8, 10.

Table s2:

Simple model with contemporary b paths for depression as mediator

Simplex for mediation with contemporary *b* path					95%CI	
Time-specific outcome	Significant Paths and Effect of treatment	Standardized estimate	SE	*p* value	Lower limit	Upper limit
Week 2 Behavioral Activation (Y_2)	Total effect	0.07	0.07	0.332	-0.06	0.20
	Indirect effect	0.02	0.02	0.386	-0.02	0.06
Week 4 Behavioral Activation (Y_3)	Total effect	0.27	0.07	<.001	0.13	0.40
	Indirect effect	0.06	0.05	0.255	-0.04	0.16
Week 6 Behavioral Activation (Y_4)	Total effect	0.28	0.07	<.001	0.14	0.41

	Indirect effect	0.24	0.06	<.001	0.12	0.35
	T->Y_3->Y_4	0.17	0.05	0.002	0.07	0.27
Week 8 Behavioral Activation (Y_5)	Total effect	0.24	0.07	0.001	0.09	0.38
	Indirect effect	0.26	0.06	<.001	0.13	0.38
	T->Y_3->Y_4->Y_5	0.14	0.05	0.003	0.06	0.24
Week 10 Behavioral Activation (End-point, Y_6)	Total effect	0.30	0.07	<0.001	0.16	0.43
	Indirect effect	0.25	0.07	0.001	0.11	0.38
	T->Y_3->Y_4->Y_5->Y_6	0.13	0.04	0.003	0.06	0.22

Note: Only significant paths were showed in the above table to save space. T = Treatment allocation (treatment group = 1, control group = 0); Y_2, Y_3, Y_4, Y_5, Y_6 = behavioral activation measurement taken at week 2, 4, 6, 8, 10.
